# Genome-Wide Identification and Expression Analysis of Zona Pellucida (ZP) Gene Family in *Cynoglossus semilaevis*

**DOI:** 10.3390/ijms26115346

**Published:** 2025-06-02

**Authors:** Kaili Zhang, Zhangfan Chen, Chengbin Gao, Xihong Li, Na Wang, Min Zhang, Haipeng Yan, Zhenxia Sha, Songlin Chen

**Affiliations:** 1 College of Life Science, Qingdao University, Qingdao 266071, China; zhangkaili@qdu.edu.cn (K.Z.); shazhenxia@163.com (Z.S.); 2 State Key Laboratory of Mariculture Biobreeding and Sustainable Goods, Yellow Sea Fisheries Research Institute, Chinese Academy of Fishery Sciences, Qingdao 266071, China; chenzf@ysfri.ac.cn (Z.C.); chengbin_gao@163.com (C.G.); lixh@ysfri.ac.cn (X.L.); wangna@ysfri.ac.cn (N.W.); 17860361038@163.com (M.Z.); yanhaipeng265438@gmail.com (H.Y.); 3 Laboratory for Marine Fisheries Science and Food Production Processes, Qingdao Marine Science and Technology Center, Qingdao 266237, China

**Keywords:** zona pellucida, *Cynoglossus semilaevis*, expression profiles, fertilization, oogenesis

## Abstract

The Chinese tongue sole (*Cynoglossus semilaevis*) is a commercially important mariculture species; however, its fertilization and hatching rates under artificial conditions remain relatively low. Zona pellucida proteins (ZPs), which mediate sperm–egg binding, were previously identified as differentially expressed genes between newly differentiated ovaries and testes in *C. semilaevis*. In this study, we identified 25 ZPs of *C. semilaevis* through genomic analysis and classified them into five subfamilies. All genes possessed a conserved ZP domain, characteristic of the gene family from mammals to teleosts. Among them, nine genes were highly expressed in ovary cells, with the expression levels increasing during ovarian development, while another three genes were predominantly expressed in liver cells. Protein–protein interaction analysis predicted that 12 ZPs interacted with key reproductive regulators such as Gdf9, Arid4a, Arid4b, and Rbl, which were involved in steroidogenesis, sperm–egg recognition, and folliculogenesis. Functional analyses using RNA interference revealed that *Cszpc7-1* knockdown in ovarian cells led to the downregulation of *cyp19a*, *esr2*, *bmp15*, and *adamts-1*, while the expression of *rbl*, *gnas*, *adgrl1*, and *adgrl2* was upregulated. In contrast, *Cszpax1* knockdown resulted in decreased expression of *cyp19a*, *foxl2*, *arid4a*, and *zeb1*, along with upregulation of *arid4b*, *ogg1*, and *gdf9*. These results suggested that ZP genes might contribute to ovarian homeostasis by regulating steroid hormone synthesis, follicular development, and ovulation. This study contributed to a deeper understanding of the reproductive mechanisms of *C. semilaevis* and provided evolutionary insights into the functional divergence of the ZP gene family across teleosts.

## 1. Introduction

In recent years, flatfish aquaculture has expanded rapidly, showing significant growth potential. However, suboptimal fertilization and hatching rates in artificial systems remain a major challenge, limiting production efficiency. For *Paralichthys lethostigma*, fertilization and hatching rates under artificial conditions are 33.30 ± 2.18% and 53.00 ± 3.00%, respectively—far lower than the 82.1 ± 1.3% and 86.1 ± 1.5% rates observed in natural environments [1]. Similarly, *Scophthalmus maximus* exhibits fertilization rates consistently below 50% in aquaculture settings [2]. These reproductive inefficiencies hinder stock production and constrain the sustainable development of commercial flatfish farming.

Fertilization is a complex process in which sperm recognition of the zona pellucida (ZP) is a critical step. The ZP, a glycoprotein-rich structure surrounding the oocyte, is composed of specialized proteins known as zona pellucida proteins (ZPs). Since the first instance of identification and characterization of ZP proteins was described in mice [3,4], ZP proteins have since been characterized in various vertebrates, including humans [5], chickens [6], and medaka [7]. Genomic analyses reveal considerable interspecies variations in ZP gene copy numbers. For instance, fish genomes contain varying numbers of ZP genes, with 21 in zebrafish *Danio rerio*, 20 in medaka *Oryzias latipes*, and 19 in Nile tilapia *Oreochromis niloticus* [8]. Unlike mammals, fish exhibit a greater expansion of ZPB, ZPC, and ZPAX subfamilies, reflecting lineage-specific diversification.

ZP proteins play essential roles in sperm–egg recognition, the induction of the acrosome reaction, and the protection of fertilized eggs and early embryos [9]. Most functional studies on ZP genes have been conducted in mammals. In mice, *ZP1* contributes to the structural integrity of the ZP matrix by linking ZP fibers [10], while *ZP2* is involved in secondary sperm binding following the acrosome reaction [11]. Mutations in *ZP3* disrupt oocyte meiosis by reducing the percentage of MII oocytes, indicating its role in germinal vesicle breakdown [12].

Compared to mammals, research on the ZP gene family in teleosts remains limited, with most studies focusing on gene expression patterns and regulatory factors. In teleost fish, ZP genes are predominantly expressed in the ovary, although some exhibit high expression in the liver. In medaka, three ZP genes are liver-expressed, while the rest are ovary-expressed [13]. Similarly, in Nile tilapia, *zpb2b* and *zpc2* are expressed in the liver and contain estrogen response elements (EREs) in their promoter regions [8]. Beyond their structural role in the egg envelope, ZP genes also perform additional functions in teleosts. For example, ovary-expressed *zp3a* in rare minnow influences egg adhesiveness and buoyancy [14]. In Antarctic notothenioid fishes, ZP proteins exhibit unique melting-promoting activity, enhancing egg survival in freezing conditions [15].

The Chinese tongue sole (*Cynoglossus semilaevis*) is a major marine aquaculture species in China, prized for its high nutritional value and desirable taste. Its commercial farming has expanded rapidly; however, reproductive challenges persist, as males exhibit weaker reproductive capacity and females produce lower-quality eggs, leading to fertilization and hatching rates below 50% [16]. A previous study identified differentially expressed genes between testes and ovaries of *C. semilaevis*, with several ZP genes displaying female-biased expression [17]. A systematic exploration of ZP genes in *C. semilaevis* was conducted through genomic survey, phylogenetic classification, and expression dynamics analysis. Additionally, the effect of ZP knockdown on genes involved in oogenesis, ovary follicle development, and ovulation was examined. Additionally, the effect of ZP genes knockdown on other genes involved in oogenesis, ovary follicle development, and ovulation was examined.

## 2. Results

### 2.1. Identification and Characterization of ZP Genes

Through domain-based screening, 25 ZP genes containing the characteristic ZP domain were identified in *C. semilaevis* (including *Cszpax1*, *Cszpax2*, *Cszpax3-1*, *Cszpax3-2*, *Cszpax4*, *Cszpax5*, *Cszpb1a*, *Cszpb1b*, *Cszpb1c*, *Cszpb1d*, *Cszpb2a*, *Cszpb2b*, *Cszpb2c*, *Cszpc2*, *Cszpc3*, *Cszpc4a*, *Cszpc4b*, *Cszpc4c*, *Cszpc5-1*, *Cszpc5-2*, *Cszpc6*, *Cszpc7-1*, *Cszpc7-2*, *Cszpc8*, and *Cszpd*). As shown in Table 1, ZP family members were located on nine different autosomes. These ZP genes exhibited open reading frame (ORF) sequences ranging from 642 bp to 3282 bp, encoding proteins of 214–1094 amino acids. MWs spanned 23.67–123.50 kDa, with pIs varying from 4.67 to 9.09.

### 2.2. Phylogenetic Analysis of ZP Family

**Figure 1:**
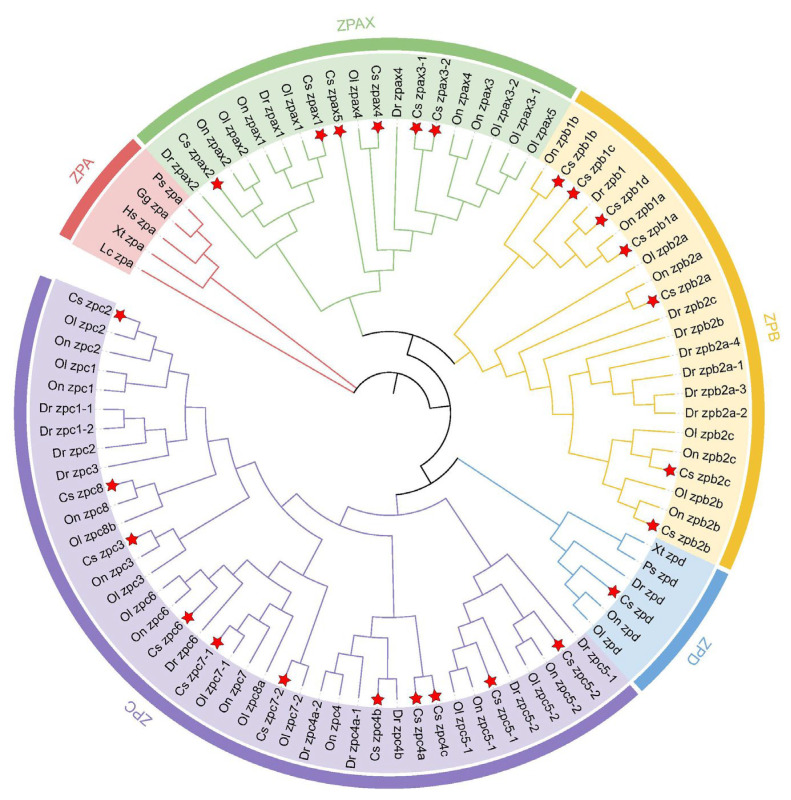
Phylogenetic analysis of ZP genes. Different colors in the circle represent different subclusters (red for ZPA, green for ZPAX, yellow for ZPB, blue for ZPD, purple for ZPC). Phylogenetic tree for nine species: *C*. *semilaevis* (Cs), *Homo sapiens* (Hs), *D. rerio* (Dr), *O. niloticus* (On), *Gallus gallus* (Gg), *X. troicalis* (Xt), *Latimeria chalumnae* (Lc), *Pelodiscus sinensis* (Ps), and *O. latipes* (Ol). The five subclusters are represented in different colors. CsZPs were marked with red stars.

**Figure A1:**
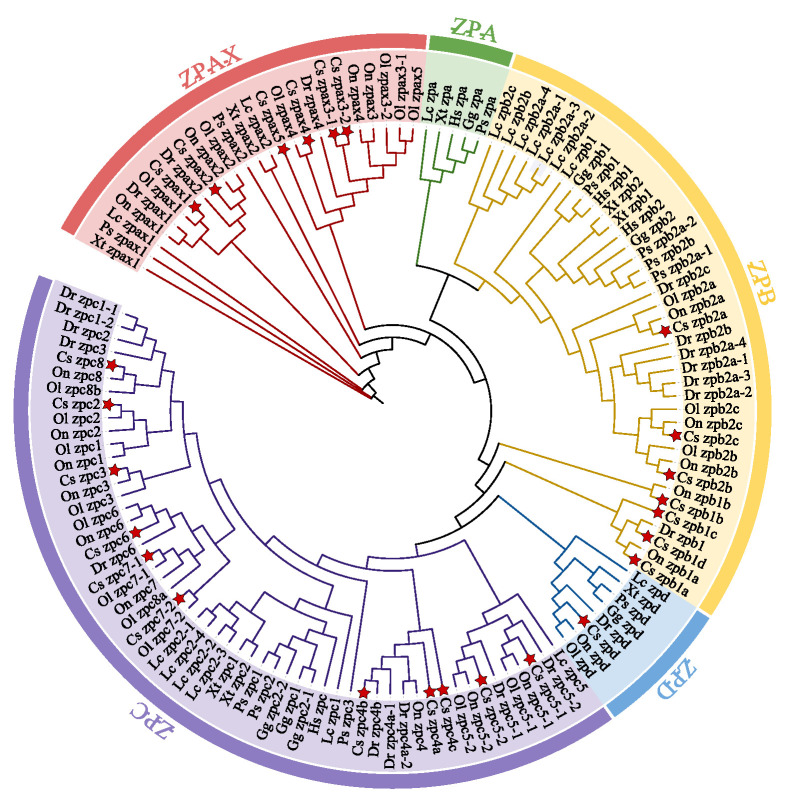
Phylogenetic tree of ZPs were constructed using all ZP protein sequences from the following species: *C. semilaevis* (Cs), *H. sapiens* (Hs), *D. rerio* (Dr), *O. niloticus* (On), *G. gallus* (Gg), *X. troicalis* (Xt), *L. chalumnae* (Lc), *P. sinensis* (Ps), and *O. latipes* (Ol). The five subclusters are represented in different colors. CsZPs are marked with red stars.

As shown in Figure 1, five distinct subclusters (ZPA, ZPAX, ZPB, ZPC, and ZPD subfamilies) were strikingly observed. Within each subfamily, amino acid sequences from different four teleost species clustered together, while the evolutionary relationships among mammals, amphibians, reptiles, and other species are shown in Appendix A Figure A1. Interestingly, the same subfamily genes from nine species were clustered in the same subcluster completely. Noteworthily, teleost fish possessed all ZP subfamilies except for ZPA. Among these subfamilies, ZPD maintained a single copy across various teleost species, whereas the ZPAX, ZPB, and ZPC subfamilies had undergone expansion. All ZP genes in *C. semilaevis* were embedded with the teleost clade, revealing a highly close relationship with other fish species.

### 2.3. Gene Structural Features of ZP Family

**Figure 2:**
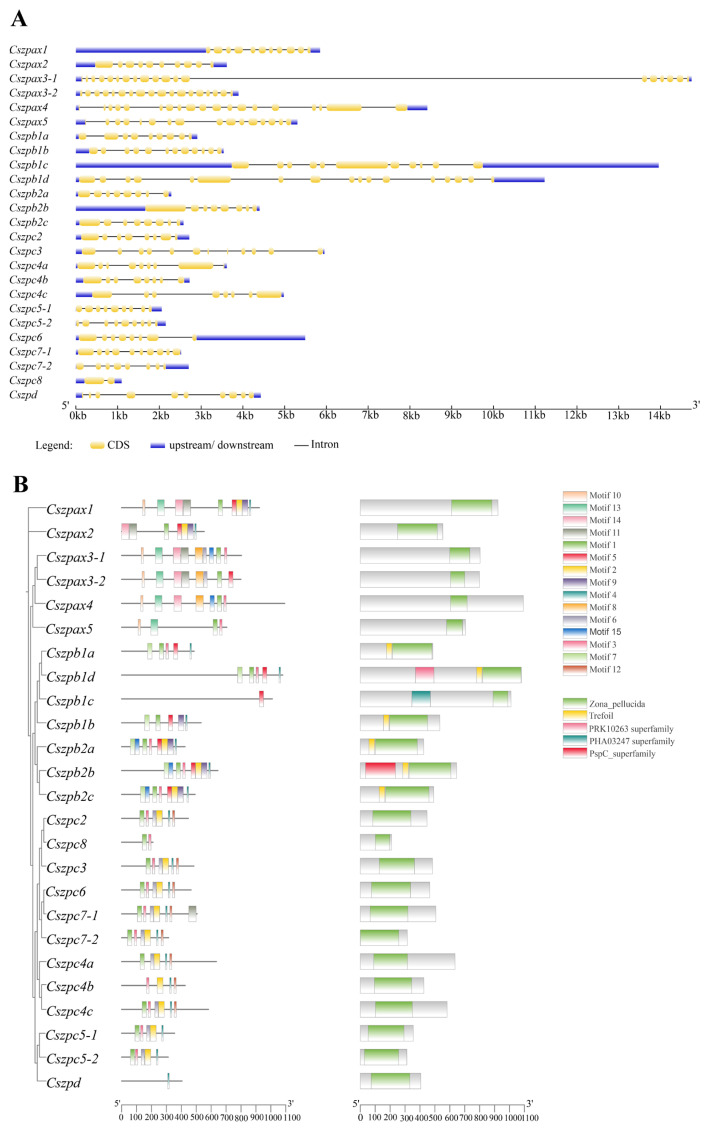
Gene structures and conserved domains of ZP family members. (**A**) Gene structures. The yellow and blue rectangles indicate the CDS and UTR regions; the gray line represents the introns. (**B**) The conserved domains of ZP family members. Different colored and shapes indicate different domains and motifs. Horizontal gray bars indicate amino acid sequences with no predictive functional domains. Protein domains are shown relative to the length of the position in the amino acid sequences.

The ZP gene family displays remarkable structural diversity, with individual genes containing between 1 and 24 introns interspersed with 2–25 exons. (Figure 2A).

As shown in Figure 2B, all proteins shared a common ZP domain, which was typically located at the C-terminus of the polypeptide. However, the specialized trefoil domain was exclusively present in the ZPB subtype.

Fifteen conserved motifs were identified in the ZP genes of *C. semilaevis*, demonstrating significant sequence conservation across the gene family. (Figure 2B). Three conserved motifs (Motif3, Motif4, and Motif1) existed in almost all members. In addition, the type and number of conserved motifs showed higher similarity between the same subfamily, suggesting that members of the same subfamily may have similar functions. Some members have unique motifs, suggesting functional variability between subfamilies.

### 2.4. Syntenic Analysis of ZP Genes in Teleost

**Figure 3:**
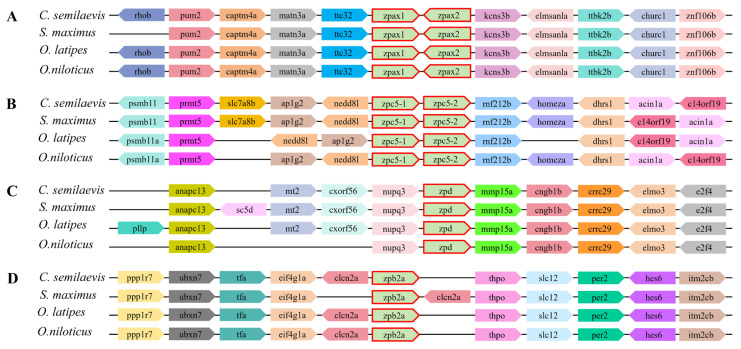
Synteny analysis of ZP gene in selected teleosts. (**A**) Synteny analysis of *zpax1* and *zpax2* genes. (**B**) Synteny analysis of *zpc5-1* and *zpc5-2* genes. (**C**) Synteny analysis of *zpd* genes. (**D**) Synteny analysis of *zpb2a* genes. The colored pentagons indicate different genes, and the direction of each pentagon indicates the gene direction. The empty space indicates a region with other genes or the absence of the gene in the genome.

To further investigate the conservation and genomic location of ZP genes with its neighboring genes, a synteny analysis was conducted between ZP genes of *C. semilaevis* and other teleost fish, including *O. niloticus*, *S. maximus*, and *O. latipes*. The results showed a high degree of conservatism (Figure 3). One to two genes from the four subfamilies of *C. semilaevis* were selected for investigation.

Similar upstream and downstream genes were present in the four fish species. In detail, the *zpax1* gene and *zpax2* gene arranged in tandem on the chromosome in *C. semilaevis* and the other three selected teleost fish species (Figure 3A), as well as *zpc5-1* and *zpc5-2* (Figure 3B). Noteworthily, there were some inversions that appeared in the adjacent linear genes of *zpc5-1* and *zpc5-2*. For example, *nedd8l* and *aplg2* were inverted in *O. niloticus*, and there was an inversion between *c14orf19* and *acin1a* in *S. maximus* and *O. latipes*. Moreover, several genes were missing or inserted upstream and downstream of these ZP genes in some fish species, especially for *zpd* and *zpb2a* (Figure 3C,D). These phenomena might indicate the evolutionary conservation between different species.

### 2.5. Protein–Protein Interaction (PPI) Network Analysis

**Figure 4:**
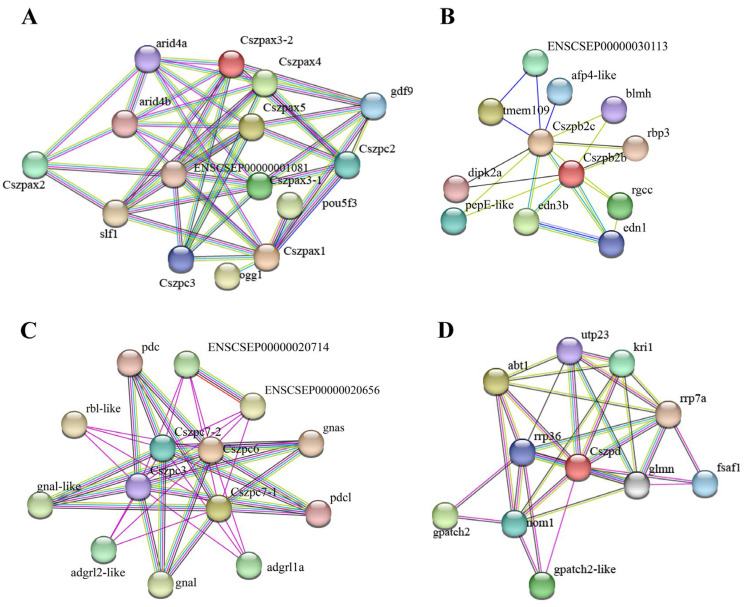
The predicted ZP genes functional partners using the protein–protein interaction method (PPI). (**A**) PPI analysis of ZPAX. (**B**) PPI analysis of ZPB. (**C**) PPI analysis of ZPC. (**D**) PPI analysis of ZPD.

To better understand the function and interaction relationships of ZP genes in *C. semilaevis*, PPI network diagrams for each subfamily were constructed (Figure 4). Based on string analysis, there were 39 functional partners that built a PPI network together with CsZPs, including 9 proteins for Zpax, 10 for Zpb, 10 for Zpc, and 10 for Zpd. Seven proteins involved in cell proliferation and cycle regulation were identified within the CsZP-associated PPI networks, including Arid4a and Arid4b in the Zpax-associated network; Dipk2a and Edn1 in the Zpb-associated network; and Gpatch, Nom1, and Glmn in the Zpd-associated network. Gdf9 proteins were the partners for Zpax only. The oxidative stress factors Ogg1 and Slf1 were found to be associated exclusively with the Zpax network, and the activator Abt1 may be related to the transcriptional regulation of Zpd.

### 2.6. Expression Patterns of ZP Genes in C. semilaevis

**Figure 5:**
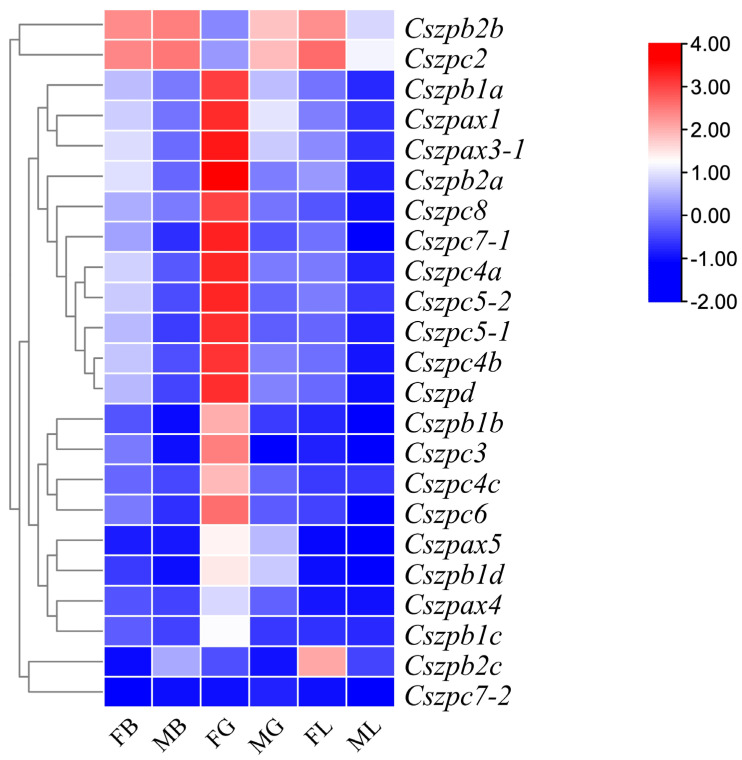
Heatmap of ZP family members; mRNA abundances in three different tissues of healthy male and female *C. semilaevis*. FB−female brain; MB−male brain; FG−female gonad; MG−male gonad; FL−female liver; ML−male liver. The expression levels were quantified as FPKM based on RNA-Seq. Gene expression levels are color coded from low (blue) to high (red). Each row represents one gene (listed on the right). Note: Only 23 of the 25 gene family members are shown in the heatmap because *Cszpax2* and *Cszpax3-1* were not detected in the transcriptome data, likely due to very low expression levels.

Comparative transcriptome analysis showed that most ZP genes exhibited significant differences in mRNA abundance across the brain, gonad, and liver tissues 1.5 years post-hatch (yph) *C. semilaevis* (Figure 5). Thirteen genes (*Cszpax1*, *Cszpax3-1*, *Cszpb1a*, *Cszpb2a*, *Cszpc3*, *Cszpc4a*, *Cszpc4b*, *Cszpc5-1*, *Cszpc5-2*, *Cszpc6*, *Cszpc7-1*, *Cszpc8*, and *Cszpd*) displayed gonad-biased expression exclusively in females, with negligible expression in all male tissues. *Cszpb2c* displayed liver-specific expression. In contrast, *Cszpb2b* and *Cszpc2* were highly expressed in the female liver while also showing moderate expression in both male and female brains. Notably, *Cszpc7-2* transcripts were undetectable across all examined tissues under the experimental conditions.

**Figure 6:**
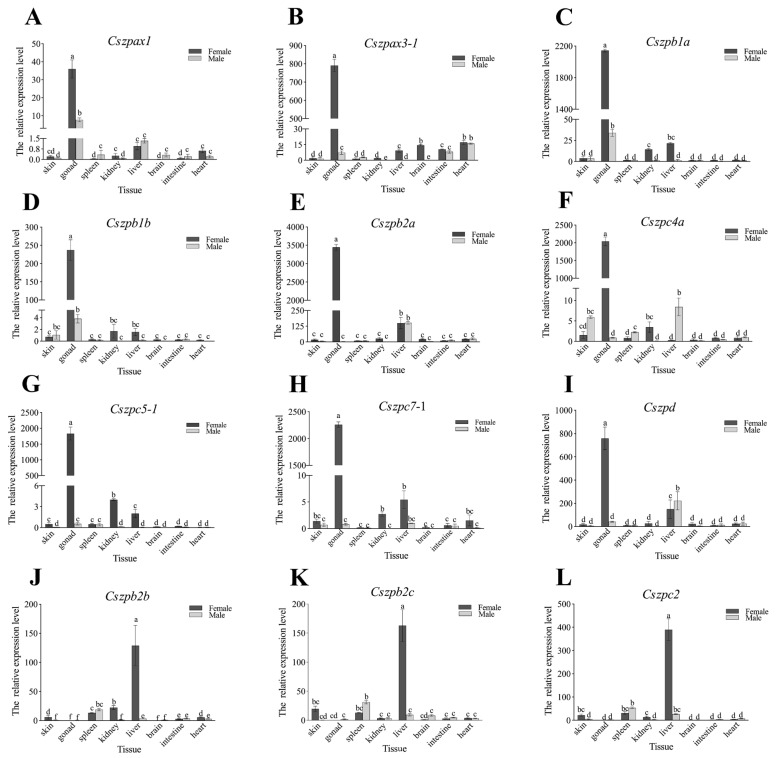
Relative expression levels of ZP genes in different tissue of male and female *C. semilaevis*. (**A**) *Cszpax1*. (**B**) *Cszpax3-1*. (**C**) *Cszpb1a*. (**D**) *Cszpb1b*. (**E**) *Cszpb2a*. (**F**) *Cszpc4a*. (**G**) *Cszpc5-1*. (**H**) *Cszpc7-1*. (**I**) *Cszpd*. (**J**) *Cszpb2b*. (**K**) *Cszpb2c*. (**L**) *Cszpc2*. The brown and gray separately represent female and male. Bars with different letters indicate statistically significant differences (*p* < 0.05).

**Figure 7:**
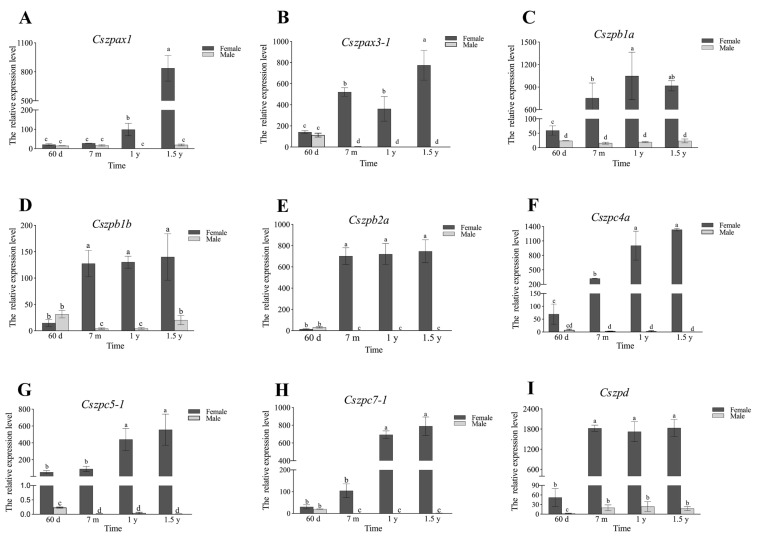
Relative expression levels of ZP genes in the gonadal tissues of male and female *C. semilaevis* at different times. (**A**) *Cszpax1*. (**B**) *Cszpax3-1*. (**C**) *Cszpb1a*. (**D**) *Cszpb1b*. (**E**) *Cszpb2a*. (**F**) *Cszpc4a*. (**G**) *Cszpc5-1*. (**H**) *Cszpc7-1*. (**I**) *Cszpd*. The brown and gray separately represent female and male. Bars with different letters indicate statistically significant differences (*p* < 0.05).

qPCR analysis further validated the transcriptomic findings (Figure 6). Nine ovary-enriched genes exhibited predominant expression exclusively in the ovaries 1.5 yph in female gonads (>100-fold higher) compared to other tissues. Conversely, three liver-and brain-enriched genes showed exceptionally high expression in the livers of 1.5 yph females (>100-fold elevation), while their expression levels in the brain samples remained comparatively low. We subsequent examined the expression trends of the nine ovary-enriched genes in gonads at different developmental stages (Figure 7). The results showed that *Cszpax1*, *Cszpax3-1*, *Cszpb1b*, *Cszpb2a*, *Cszpc4a*, and *Cszpc7-1* did not exhibit sexual-biased expression in 60-day post-hatch (dph) individuals but displayed significantly higher expression in ovaries compared to testes from the 7-month post-hatch (mph) to 1 yph individuals. The expressions of almost all genes reached their peaks in the ovaries at 1.5 yph.

**Figure 8:**
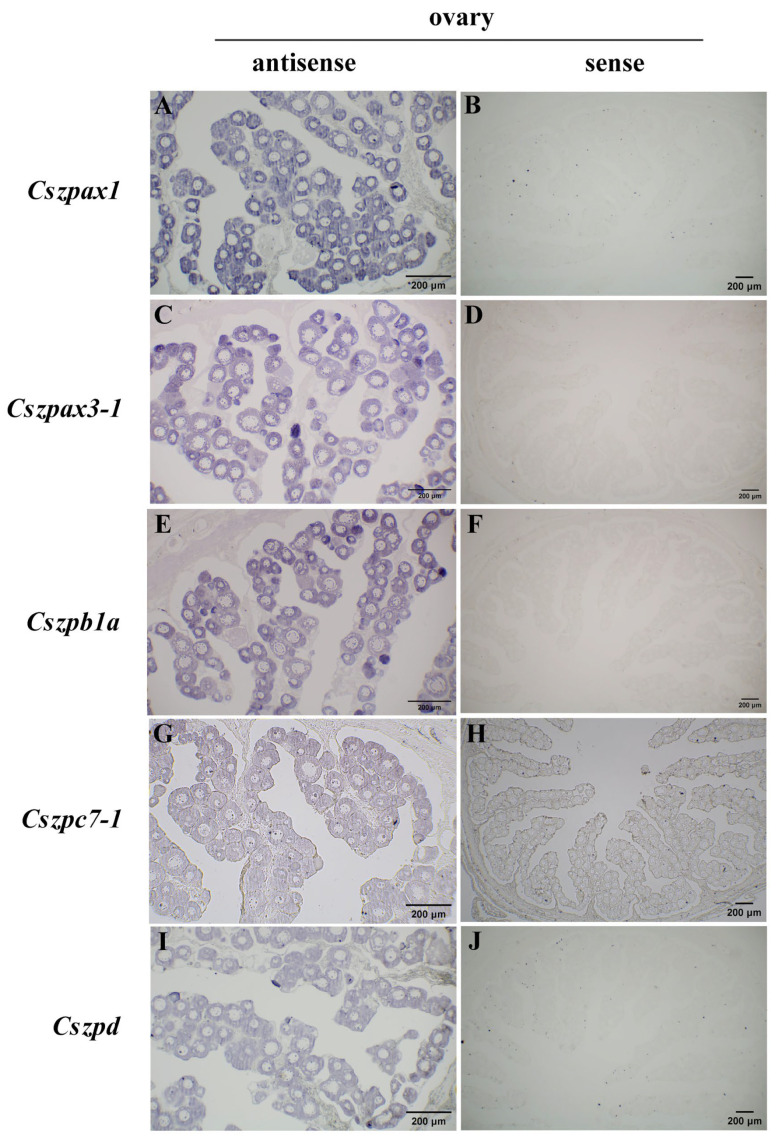
The spatial expression patterns of ZP genes of *C. semilaevis* at 1.5 yph. (**A**) Antisense probe for *Cszpax1* in ovary. (**B**) Sense probe for *Cszpax1* in ovary. (**C**) Antisense probe for *Cszpax3-1* in ovary. (**D**) Sense probe for *Cszpax3-1* in ovary. (**E**) Antisense probe for *Cszpb1a* in ovary. (**F**) Sense probe for *Cszpb1a* in ovary. (**G**) Antisense probe for *Cszpc7-1 in ovary*. (**H**) Sense probe for *Cszpc7-1* in ovary. (**I**) Antisense probe for *Cszpd* in ovary. (**J**) Sense probe for *Cszpd* in ovary. Strong hybridization signals of *Cszpax1*, *Cszpax3-1*, *Cszpb1a*, *Cszpc7-1*, and *Cszpd* were specifically localized in the oocytes of female gonadal tissues. Scale bars = 200 μm.

In the ovaries of 1.5 yph individuals, we detected strong hybridization signals for *Cszpax1*, *Cszpax3-1*, *Cszpb1a*, *Cszpc7-1*, and *Cszpd* genes, while no detectable signals were observed in the testes (Figure 8). This result is consistent with transcriptomic and qPCR results, and these genes exhibited expression throughout all developmental stages of oocytes within the ovaries.

### 2.7. Knockdown Effects of Cszpax1, Cszpc7-1 on CO

**Figure 9:**
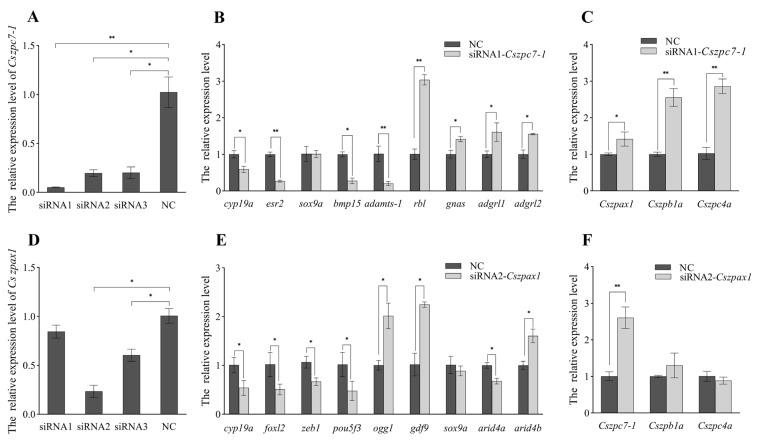
The knockdown effects of *Cszpc7-1* and *Cszpax1* siRNA in CO cells. (**A**–**C**). Knockdown efficiency of *Cszpc7-1* and its effects on sex-related genes and other ZP genes. (**D**–**F**). Knockdown efficiency of *Cszpax1* and its effects on sex-related genes and other ZP genes. A *p*-value less than 0.05 was considered to indicate a significant difference between the two groups and indicated by *. (*—*p* < 0.05; **—*p* < 0.01).

Three siRNA targeting specific sites of *Cszpax1* and *Cszpc7-1* were designed to knockdown gene expression in CO. The expression levels of *Cszpax1* and *Cszpc7-1* decreased significantly by 80% and 60%, with siRNA1-*Cszpc7* and siRNA2-*Cszpax1* showing the best knockdown effects (Figure 9). In the subsequent RNA interference (RNAi) experiments, the expressions of genes associated with sex determination and ovarian development were affected. Following *Cszpc7-1* knockdown, the expression levels of *cyp19a*, *esr2*, *adamts-1*, and *bmp15* were significantly downregulated, while the expressions of *rbl*, *gnas*, *adgrl1*, and *adgrl2* were markedly upregulated. After *Cszpax1* knockdown, the expression levels of *foxl2*, *zeb1*, *arid4a*, and *cyp19a* were markedly downregulated. In contrast, the expression of *ogg1*, *gdf9*, and *arid4b* was notably upregulated. However, *sox9a* gene expression did not chance significantly. Additionally, *Cszpc7-1* knockdown resulted in upregulation of other ZP genes such as *Cszpb1a*, *Cszpax1*, and *Cszpc4a*, while *Cszpax1* knockdown only led to a significant increase in *Cszpc7-1* expression.

## 3. Discussion

In teleosts, zona pellucida genes contribute not only to sperm-egg recognition but to even broader functions, regulating egg adhesiveness and buoyancy [14], as well as enhancing egg antifreeze properties during cold conditions [15]. All known ZP genes contain a conserved ZP domain at the C-terminus, which is likely critical for the biological function of ZP proteins. Through genome-wide screening for candidate sequences containing the ZP domain and subsequent phylogenetic analysis, we identified 25 ZP genes in the *C. semilaevis* genome, including seven ZPB genes, eleven ZPC genes, one ZPD gene, and six ZPAX genes. Fish proteins have evolved significantly faster than their mammalian homologues due to the presence of genome duplication events in fish [18,19]. Gene duplication led to the creation of the ancestors of the various subfamilies of ZP, and thus, fish ZP genes tend to be multiply replicated [20], with the ZPC gene being abundantly replicated in zebrafish and medaka [21].

Interestingly, the *C. semilaevis* genome lacks ZPA genes, which is consistent with findings in other teleosts [20,22,23]. Compared to the human genome, the ZPB, ZPC, and ZPAX subfamilies have undergone significant expansion in teleosts but still beyond a high degree of evolutionary conservation. The *C. semilaevis* genome contains seven ZPB genes, with six of them (excluding *Cszpb1c*) possessing a trefoil domain. This unique three-loop structure, stabilized by disulfide bonds between six conserved cysteine residues, functions as a structural component of the zona pellucida rather than as a sperm receptor [24]. While the chicken genome contains only two ZPAX genes, teleost genomes have a larger number, with six ZPAX genes identified in *C. semilaevis*. Similarly, the ZPC subfamily has also expanded in *C. semilaevis*, reaching a total of eleven genes. Goudet et al. proposed that species relying on external fertilization might require a larger repertoire of ZP genes [21]. Compared to mammals, fish eggs have a larger radius and are released in large quantities, which may necessitate a greater number of egg envelope proteins [25]. Moreover, teleost fish eggs are exposed to more challenging conditions, and the egg envelope hardens during fertilization, providing mechanical protection against external pressures and bacterial infections [26,27]. These environmental and reproductive pressures likely drove the expansion of the ZP gene family in teleosts, with multiple ZP genes playing critical roles in fertilization and embryonic development.

Previous study revealed nine ZP genes with ovary-biased expression and three ZP genes with liver-biased expression in the *C. semilaevis* transcriptome [17]. In this study, qPCR results confirmed that nine ovary-expressed ZP genes were upregulated hundreds of times compared with their expressions in other examined tissues. Further, *Cszpb1a*, *Cszpc5-1*, and *Cszpd* already exhibited female-biased expression at the early stage of gonadal differentiation (60 dph). As development progressed and the gonads matured, all nine genes accumulated in the ovaries of 1.5 yph individuals, with consistent expression in oocytes at all developmental stages. These findings indicate that ovary-specific ZP genes play a crucial role in ovarian development and maturation. In medaka, *zpb2b*, *zpb2c*, and *zpc2* are highly expressed in the liver [7,28]. Similarly, in tilapia, *zpb2b* and *zpc2* exhibit predominant expression in the liver [8]. The *zpb2b*, *zpb2c*, and *zpc2* in *C. semilaevis*, which cluster phylogenetically with these genes, also show significant liver expression. This suggests that during evolution, the teleost has developed lineage-specific adaptations in ZP gene expression, with some species expressing ZP genes in either the ovary or liver. Salmon eggs have a relatively thick chorion (30~40 μm), and their ZP genes are expressed in both the liver and ovary [29]. In contrast, zebrafish eggs have a much thinner chorion (~5 μm), which may require fewer ZP products, leading to the exclusive ovarian expression of ZP genes [30]. Although *C. semilaevis* also has a relatively thin chorion, the open marine environment differs significantly from freshwater habitats. Most marine fish produce pelagic eggs with oil droplets and lack parental egg-guarding behaviors. Additionally, marine fish generally have higher fecundity than freshwater species, necessitating the production of more egg envelope proteins. *Zeb1* is known to regulate germ cell mitosis and gametogenesis in mice [31]. In *C. semilaevis*, *zeb1* is significantly enriched in the pseudotemporal differentiation trajectory, suggesting its potential to promote gamete maturation through chromatin remodeling or epigenetic modifications [32]. The knockdown of *Cszpax1* led to a reduction in *zeb1* expression, which may affect oocyte maturation and ovulation, although the precise mechanism requires further investigation.

To construct the interaction networks for CsZps, we employed PPI network analysis and RNAi. When *Cszpc7-1* expression was reduced, the expression of *rbl*, *gnas*, *adgrl1*, and *adgrl2* was upregulated. Rbl proteins in fish eggs prevent polysperm entry by associating with cortical granules [33,34]. Upregulated *rbl* expression following *Cszpc7-1* knockdown implies the involvement of the ZP gene in cortical-granule-mediated polysperm blockade. G protein-coupled receptors (GPCRs) are closely related to oocyte maturation in mammals and fish [35,36,37]. As members of GPCRs, *gnas*, *adgrl1*, and *adgrl2* were found to be upregulated with *Cszpc7-1* knockdown, suggesting that *Cszpc7-1* might participate in oocyte maturation via GPCR signaling regulation. When *Cszpax1* expression was reduced, the expression levels of *arid4a* and *pou5f3* were downregulated. In contrast, the expression levels of *ogg1*, *gdf9*, and *arid4b* was upregulated. The ARID gene family, which encodes transcriptional regulators, plays essential roles in cell differentiation and proliferation [38]. *Arid4a*^−/−^ *Arid4b*^+/−^ mice exhibited reduced fertility, Sertoli cell dysfunction, and spermatogenic failure [39]. *Pou5f3* (*oct4*), a potential key regulator of mouse oocyte development [40], plays a post-embryonic role during the maturation of gonads and gametes in medaka [41]. *Gdf9* has been reported to play roles in ovarian folliculogenesis and ovulation in mammalian species [42]. It also regulates *C. semilaevis* reproduction and growth via the Smad signaling pathway [43]. *Ogg1*, the first enzyme in the base excision repair pathway, is involved in protecting oocytes against oxidative stress post-fertilization [44]. Taken together, the knockdown effects of *Cszpc7-1* and *Cszpax1* genes confirm their PPI network and suggest their potential function in oocyte development and protection.

On another hand, we examined the expression of a number of genes associated with sex determination and ovarian development. When *Cszpc7-1* expression was reduced, the expression levels of several genes involved in ovary development, including *cyp19a*, *esr2*, *bmp15*, and *adamts-1*, also decreased. Similarly, when *Cszpax1* expression was reduced, numerous ovarian-development-related genes including *cyp19a*, *foxl2*, and *zeb1*, were downregulated, while the spermatogenesis-related gene *sox9a* remained unaffected. Aromatase *cyp19a*, cooperating with *foxl2*, plays a crucial role in the biosynthesis of gonadal steroids, as well as ovarian differentiation oocyte maturation and in teleosts [45,46,47]. In zebrafish, the loss of *foxl2* leads to *cyp19a* downregulation, reduced estrogen levels, and oocyte apoptosis [48].In addition, *esr2*, a member of the estrogen receptor family, is regulated by *cyp19a* and binds to estradiol (E2) to facilitate oocyte maturation and ovulation [49]. In *C. semilaevis*, the downregulation of *foxl2*, *cyp19a*, and *esr2* with CsZP knockdown implies that CsZPs might maintain ovarian function through estrogen synthesis and signaling pathways. *Bmp15*, a member of the TGF-β superfamily, collaborates with *gdf9* to regulate folliculogenesis and steroidogenesis in mammals. Knockout of these two genes in mice leads to reduced fertility or even infertility [50]. *Adamts-1* is critical for follicular growth and ovulation in mice, and its deficiency lowers ovulation rates and fertilization efficiency [51,52,53]. Our results reveal the potential function of CsZP genes in oocyte development and fertilization, which is consistent with mammalian studies demonstrating the essential role of ZP genes in these processes, where their knockouts leads to infertility or impaired folliculogenesis [12,54,55,56,57].

We also investigated the effects of *Cszpax1* and *Cszpc7-1* knockdown on the expression of other ZP genes and found a compensatory expression effect between *Cszpax1* and *Cszpc7-1*. This suggests that ovarian cells may respond to ZP gene knockdown by modulating gene expression levels to mitigate or counteract the effects of RNAi interference on the ZP gene interaction network.

## 4. Materials and Methods

### 4.1. Animal Euthanasia and Ethics Statement

In this study, fish were anesthetized using MS-222 (Solarbio, Beijing, China)(120 mg/L) to minimize pain. All experimental adhered to the guidelines established by the Institutional Animal Care and Use Committee of the Yellow Sea Fisheries Research Institute.

### 4.2. Fish and Sample Collection

Fish samples in this study were collected from Haiyang Aquaculture Base (Haiyang, China). Genetic sex determination was performed by excising tall fin clips from each individual and subjecting them to PCR-based assays using established sex-specific primers (Table A1) [58]. After dissection, tissues were collected from four female and four male 1.5 yph *C. semilaevis*, including skin, gonad, spleen, kidney, liver, brain, intestine, and heart. Additionally, gonadal tissues from four different developmental stages (including 60 dph, 7 mph, 1 yph, and 1.5 yph) were collected. These tissues were flash-frozen in liquid nitrogen and maintained at −80 °C. Total RNA was isolated from tissue samples using TRIzol reagent (Invitrogen, Carlsbad, CA, USA) and then reverse transcribed to cDNA using ReverTra Ace qPCR RT Master Mix (Toyobo, Osaka, Japan) and stored at −20 °C. Gonadal tissues of 1.5 yph fish were also fixed in 4% paraformaldehyde (Solarbio, Beijing, China) at 4 °C for 24 h, embedded in paraffin, and sectioned for in situ hybridization (ISH) analysis.

### 4.3. Identification of ZP Genes in C. semilaevis Genome

To identify all ZP gene members, the data corresponding to ZP domain (PF00100) were downloaded from the PFAM protein family database (version 35, EMBL-EBI, Hinxton, Cambridgeshire, UK) as a seed [59]. Then, HMMER software (version 3.0, HMMER.org, Howard Hughes Medical Institute, Chevy Chase, MA, USA) was employed to construct a Hidden Markov Model (HMM) [60], which was used to identify the superfamily members from *C. semilaevis* genome. The amino acid and nucleotide sequences of the homologous ZP genes from human (*H. sapiens*), chicken (*Gallus gallus*), turtle (*Pelodiscus sinensis*), Xenopus (*X. tropicalis*), Coelacanth (*Latimeria chalumnae*), Nile tilapia (*O. niloticus*), zebrafish (*D. rerio*), and medaka (*O. latipes*) were downloaded from the National Center for Biotechnology Information (NCBI) database (https://www.ncbi.nlm.nih.gov/, accessed on 7 October 2023) and Ensembl databases (http://asia.ensembl.org/index.html, accessed on 19 October 2023), and the relevant information was listed in Table A2. *C. semilaevis* ZP gene candidates were captured in the transcriptome and genome databases using the BLAST+ software (version 2.12.0, National Center for Biotechnology Information, Bethesda, MA, USA) [61,62] against the homologous ZP gene sequences from other species, with a cutoff E-value of 1 × 10^−10^e^−10^. The final ZP genes of *C. semilaevis* were confirmed by combining the results from the HMMER and BLAST searches.

### 4.4. Sequence and Evolutionary Analysis

To obtain the phylogenetic relationships of ZP genes in teleost fish, ZP gene family sequences from various species were used (Table A2). Multiple sequence alignment (MSA) (version 7.505, Computational Biology Research Center, AIST, Tokyo, Japan) was conducted using MAFFT with default parameters to align the amino acid sequences [63]. Then, a phylogenetic tree was constructed using the maximum likelihood method via IQ-TREE 2 software (version 2.4.0, Center for Integrative Bioinformatics Vienna, Vienna, Austria) [64]. Next, the tree file generated by IQ-TREE was uploaded to iTol (version 5, European Molecular Biology Laboratory, Heidelberg, Germany) for visualization and customization [65]. The exon/intron gene structures of *C. semilaevis* sequence were analyzed using Gene structure display server 2.0 (GSDS 2.0, version 2.0, Center for Bioinformatics, Peking University, Beijing, China) [66]. The conserved structural domains and motifs were characterized using PFAM database and MEME program (version 5.4.1, MEME Suite, National Institutes of Health, Bethesda, MA, USA) [67], followed by visualization using the TBtools software (version 2.142, South China Agricultural University, Guangzhou, China) [68]. The chromosomal positions of sequences were obtained from the NCBI database. The molecular masses (MWs) and theoretical isoelectric points (pIs) were predicted by ExPASy (https://web.expasy.org/protparam/, accessed on 19 October 2023).

### 4.5. Synteny Analysis

Synteny analysis of ZP genes in teleost fish, including *C. semilaevis*, *O. niloticus*, *S. maximus*, *O. latipes*, was performed. The genomic annotation information was obtained from the Ensembl databases. Syntenic regions near the ZP genes were identified using the Genomicus database (version 100.01, Institut de Biologie de l’École Normale Supérieure, Paris, France) following the method previously described by Zhu et al. [69,70]. For each ZP gene, five neighboring genes upstream and five downstream genes were selected for comparison.

### 4.6. Protein–Protein Interaction Prediction

Based on the homology of *C. semiaevis*, the Search Tool for The Retrieval of Interacting Genes/Proteins (STRING) database (version 11.5, European Molecular Biology Laboratory, Heidelberg, Germany) was used to predict the potential role partners of the proteins with medium confidence (0.40) [71].

### 4.7. Heatmap Plotting with RNA-Seq Data

We utilized previous RNA-seq data of the gonad, brain, and liver samples from 1.5 yph fish [17] to understand the mRNA distribution of ZP genes in *C. semilaevis*. The heatmap was generated using the TBtools software to visualize the mRNA expression profile.

### 4.8. Quantitative Real-Time (qPCR) Analysis

The primers used for qPCR are listed in Table A1. Actin was selected as the internal reference gene. Reactions were completed using THUNDERBIRD™ Next SYBR^®^ qPCR Mix (Toyobo, Osaka, Japan) and 7500 Fast Real-Time PCR system (ABI, Los Angeles, CA, USA). Amplification conditions were 95 °C for 30 s, 95 °C for 5 s, 60 °C for 34 s, and 40 cycles. Each set of samples was repeated four times. The experimental data were obtained using the 2^−ΔΔCt^ method [72]. Data were analyzed by one-way ANOVA using SPSS version 20.0 (IBM, Armonk, NY, USA), followed by Tukey’s post hoc test for multiple comparisons. A *p*-value < 0.05 was considered statistically significant.

### 4.9. In Situ Hybridization Analysis

The primers used in this experiment to amplify fragments of *Cszpax1*, *Cszpax3-1*, *Cszpb1a*, *Cszpc7-1*, and *Cszpd* are listed in Table A1. T7/SP6 RNA polymerase promoters were introduced for in vitro synthesis of antisense/sense probe. After synthesized of the probes using the digoxigenin (DIG) RNA labeling kit (Roche Diagnostics, Nutley, NJ, USA), the probes were purified were purified using the lithium chloride (LiCl) (Sigma, Darmstadt, Germany) precipitation method for the next hybridization. In situ hybridization procedure followed the protocol outlined by Zhu et al. [73]. Signals were detected using the BCIP/NBT substrate (Roche, Mannheim, Germany) and pictures were acquired with a Nikon ECLIPSE 80i microscope (Nikon, Tokyo, Japan).

### 4.10. Cell Culture and siRNA Transfection Analysis

The *C. semilaevis* ovarian (CO) cells were cultured in L-15 medium with the following components required for cell growth: EGF (Beyotime, Shanghai, China) (5 ng/mL), bFGF (Beyotime, Shanghai, China) (5 ng/mL), LIF (Beyotime, Shanghai, China) (5 ng/mL), 2% penicillin–streptomycin–amphotericin B mixed solution (Solarbio, Beijing, China), β-Mercaptoethanol (27.5 µg/mL) (Vwr, Radnor, PA, USA), and 20% fetal bovine serum (FBS) (Gibco, Grand Island, NY, USA), and they were maintained in a 24 °C incubator without CO_2_. When the cell density reached 70–80%, the cells were inoculated in 12-well cell culture plates for transfection.

Three specific small interfering RNA (siRNA) sites of *Cszpc7-1* and *Cszpax1* were designed by Sangon (Shanghai, China) (Table A1). Targeted siRNAs and negative control (NC) samples were transfected into CO by using RiboFECT™ CP Transfection Kit (Ribobio, Guangzhou, China). Cy3-siRNA (Ribobio, Guangzhou, China) was transfected into CO at the same time for the valuation of transfection efficiency. Cells were harvest by TRIzol, and the subsequent qPCR assays were conducted as mentioned above. The expression levels of *Cszpc7-1*, *Cszpax1*, and other sex- and hormone-related genes (including *foxl2*, *cyp19a*, *sox9a*, *bmp15*, *esr2*, *adamts-1*, *rbl*, *gnas*, *adgrl1*, *adgrl2*, *pou5f3*, *gdf9*, *ogg1*, *arid4a*, *arid4b*, and *zeb1*) were examined with the primers listed in Table A1.

## 5. Conclusions

In conclusion, we identified 25 genes in the *C. semilaevis* ZP gene family, all harboring a conserved ZP domain. We analyzed the sequences and phylogeny of ZP genes and predicted their potential protein–protein interaction partners. Furthermore, we investigated the spatiotemporal expression patterns of 12 ZP genes across eight different tissues, including nine ovary-expressed and three liver-expressed members. Additionally, we analyzed the gonadal expression profiles of nine ovary-specific ZP genes during different developmental stages. *Cszpax1-* and *Cszpc7-1*-specific RNAi indicated that ZP genes might play crucial roles in oogenesis, follicular development, and ovulation, suggesting that their function is conserved and analogous to the role of ZP genes in mammals. Collectively, these findings provide valuable information for better understanding the evolution and functional diversification of ZP genes family in teleosts.

## Figures and Tables

**Table 1:** Sequence feature of ZP family members.

Name	Gene ID	Gene Length (bp)	ORF Length (bp)	Amino Acid Length (aa)	MW (kDa)	pI	Chr	Location	No. of Exons	No. of Introns
*Cszpa* *x1*	103380918	5850	2850	950	103.73	4.84	7	5,983,260–5,989,109	21	20
*Cszpa* *x2*	112487247	3614	1662	554	61.72	5.03	7	5,977,607–5,981,220	13	12
*Cszpa* *x3-1*	103386630	14,745	2412	804	91.44	5.19	1	23,962,096– 23,976,840	25	24
*Cszpa* *x3-2*	112487726	3896	2400	800	89.83	5.44	1	23,966,848–23,970,743	19	18
*Cszpa* *x4*	103380637	8419	3282	1094	123.50	6.67	7	1,154,026–1,162,444	20	19
*Cszpa* *x5*	103386730	5305	2118	706	79.67	5.78	1	23,976,991–23,982,295	16	15
*Cszpb* *1a*	103394278	2910	1461	487	54.12	4.67	18	9,161,710–9,164,619	10	9
*Cszpb* *1b*	103393374	3542	1599	533	59.41	8.84	17	12,633,779–12,637,320	12	11
*Cszpb1c*	103393397	13,959	3033	1011	110.60	5.32	17	12813072–12,827,030	16	15
*Cszpb1d*	103383792	11,226	3243	1081	120.00	6.1	9	9,156,026–9,167,251	18	17
*Cszpb2a*	103396071	2286	1278	426	47.19	5.39	20	4,011,277–4,013,562	8	7
*Cszpb2b*	103380189	4402	1938	646	72.52	5.79	6	11,810,105–11,814,506	8	7
*Cszpb2c*	103380137	2580	1479	493	52.91	7.34	6	11,208,465–11,211,044	8	7
*Cszpc2*	103380130	2718	1344	448	48.56	5.35	6	11,212,333–11,215,050	8	7
*Cszpc3*	103389891	5953	1455	485	54.01	6.11	14	17,172,577–17,178,529	12	11
*Cszpc4a*	103383075	3615	1908	636	70.82	6.19	1	2,597,477–2,601,091	9	8
*Cszpc4b*	103384013	2723	1281	427	47.76	8.65	9	13,824,630–13,827,352	9	8
*Cszpc4c*	103391190	4981	1749	583	64.97	5.79	1	4,104,869–4,109,849	8	7
*Cszpc5-1*	103396600	2058	1068	356	39.68	8.18	20	11,811,312–11,813,369	9	8
*Cszpc5-2*	103396601	2153	939	313	34.77	5.44	20	11,814,022–11,816,174	10	9
*Cszpc6*	103396037	5493	1401	467	52.55	9.09	20	3,517,755–3,523,247	9	8
*Cszpc7-1*	103392929	2522	1557	519	56.42	4.98	17	6,833,291–6,835,812	10	9
*Cszpc7-2*	103391061	2702	948	316	34.97	6.41	1	32,117,282–32,119,983	8	7
*Cszpc8*	103377641	1096	642	214	23.67	6.59	4	2,417,479–2,418,574	2	1
*Cszpd*	103379554	4431	1218	406	45.21	6.55	6	2,033,303–2,037,733	10	9

**Figure A2:**
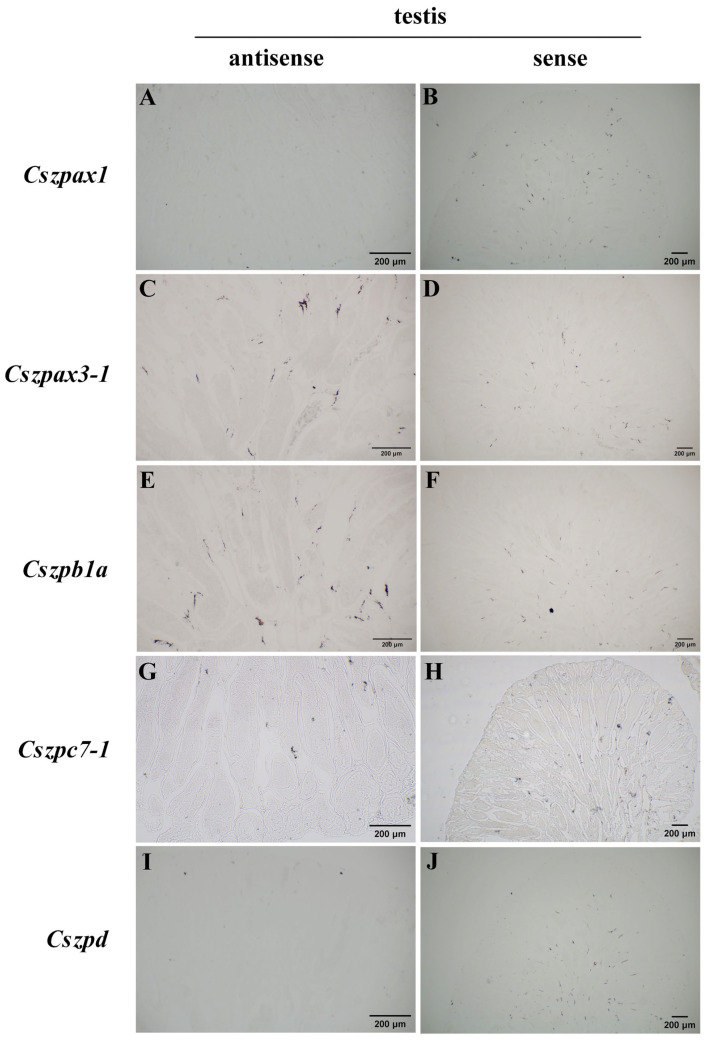
The spatial expression patterns of ZP genes of *C. semilaevis* at 1.5 yph. (**A**) Antisense probe for *Cszpax1* in testis. (**B**) Sense probe for *Cszpax1* in testis. (**C**) Antisense probe for *Cszpax3-1* in testis. (**D**) Sense probe for *Cszpax3-1* in testis. (**E**) Antisense probe for *Cszpb1a* in testis. (**F**) Sense probe for *Cszpb1a* in testis. (**G**) Antisense probe for *Cszpc7-1* in testis. (**H**) Sense probe for *Cszpc7-1* in testis. (**I**) Antisense probe for *Cszpd* in testis. (**J**) Sense probe for *Cszpd* in testis. *Cszpax1*, *Cszpax3-1*, *Cszpb1a*, *Cszpc7-1*, and *Cszpd* genes with no significant hybridization signals were observed in male gonads. Scale bars = 200 μm.

## Data Availability

The original contributions presented in this study are included in the article. Further inquiries can be directed to the corresponding author.

## Abbreviations

The following abbreviations are used in this manuscript:

*cyp19a*	cytochrome p450 19a
*foxl2*	forkhead box L2
*esr2*	estrogen receptor 2
*bmp15*	bone morphogenetic protein 15
*adamts-1*	ADAM metallopeptidase with thrombospondin type 1 motif 1
*sox9a*	SRY-box transcription factor 9a
*zeb1*	zinc finger E-box bingding homeobox 1
*rbl*	rhamnose-binding lectin
*gnas*	guanine nucleotide binding protein, alpha stimulating
*gdf9*	growth differentiation factor 9
*pou5f3*	POU class 5 homeobox 3
*adgrl1*	adhesion G protein-coupled receptor L1
*adgrl2*	adhesion G protein-coupled receptor L2
*gnal*	guanine nucleotide-binding protein G(s) subunit alpha-like
*ogg1*	8-oxoguanine DNA glycosylase 1
*arid4a*	AT-rich interaction domain 4A
*arid4b*	AT-rich interaction domain 4B

## Appendix A

**Table A1:** The primers used in the present study.

Primer	Purpose	Sequences (5′→3′)
*zpb2a*-q-F	qPCR	GCACCAGGGATGGACAGTTT
*zpb2a*-q-R	qPCR	AGGAGGGGTCATTAGGGTCC
*zpb1b*-q-F	qPCR	CTCGGTGTGGTTGCTTCTCT
*zpb1b*-q-R	qPCR	CTTGGGAGTGGGAAGTGGTC
*zpb1a*-q-F	qPCR	CCCAAAAGCAACACTTCGGG
*zpb1a*-q-R	qPCR	GGGTCTAAAGGGTTGGCACA
*zpax1*-q-F	qPCR	TGTCCAGGAGCACACGTTTT
*zpax1*-q-R	qPCR	AGAGGCAGGAAGTAGACCGT
*zpd*-q-F	qPCR	ATTGCCATCAGAGCCGTTCA
*zpd*-q-R	qPCR	CTTGGAGACGCTGGACATGT
*zpax3-1*-q-F	qPCR	CCGTATGGACAGCCTGACTC
*zpax3-1*-q-R	qPCR	CCATGATGACCACCCAGCTT
*zpc4a*-q-F	qPCR	TCAGTACCGGCCCTTTTGTC
*zpc4a*-q-R	qPCR	CCCTCATTACAGCAGCACCA
*zpc5-1*-q-F	qPCR	AATGGAGGAGTGTGTGGCAG
*zpc5-1*-q-R	qPCR	ATGGCAGATGAGTGGTAGCG
*zpc7-1*-q-F	qPCR	AACCGGAACAAACCCACAGT
*zpc7-1*-q-R	qPCR	ACAACTGGTCCCACTCTTGC
β-actin-F	qPCR	TCAATTCTCCACGAACCA
β-actin-R	qPCR	CTTACACAGCGAGCAACC
*cyp19a*-q-F	qPCR	GGTGAGGATGTGACCCAGTGT
*cyp19a*-q-R	qPCR	ACGGGCTGAAATCGCAAG
*foxl2*-q-F	qPCR	GAGAGGAAGGGCAACTACTGGA
*foxl2*-q-R	qPCR	TGGTTGGAAGTGCGTGGG
*sox9a*-q-F	qPCR	AAGAACCACACAGATCAAGACAGA
*sox9a*-q-R	qPCR	TAGTCATACTGTGCTCTGGTGATG
*bmp15*-q-F	qPCR	CGGACGGAAGAACTAAACA
*bmp15*-q-R	qPCR	TCATACTGGACCTGGAACG
*esr2*-q-F	qPCR	GATTAGGAGAAGGTGGAGAAGG
*esr2*-q-R	qPCR	GGTAACCAGAGGCATAGTCGTG
*zeb1*-q-F	qPCR	GCTCCTCTTCAAGGCACAGT
*zeb1*-q-R	qPCR	GACGTTACCATCCACTGCCA
*adamts-1*-q-F	qPCR	CTGGCCTCAAACCCTACCTG
*adamts-1*-q-R	qPCR	GGCCTCGCTCCTCTTCATAC
*zpax1*-siRNA-1	siRNA	CCAGAGUCCUUCAGAUAUA
*zpax1*-siRNA-2	siRNA	CGACUGUGCCGGCAAUCUA
*zpax1*-siRNA-3	siRNA	CAGACAAGCAGUGGUUUAA
*zpc7-1*-siRNA-1	siRNA	GGCUGUGUAGCUACGUUAA
*zpc7-1*-siRNA-2	siRNA	CGCUGGAGAUCGUCAUCAA
*zpc7-1*-siRNA-3	siRNA	CGCGAUUGUUGUCGGGCUA
*zpb1a*-SP6	ISH	ATTTAGGTGACACTATAGAATCGAGGCTACGAGGTGGATT
*zpb1a*-T7	ISH	TAATACGACTCACTATAGGGGCTCATAAACACGGGCTCCA
*zpd*-SP6	ISH	ATTTAGGTGACACTATAGAATGTGGCAGGTGTAGGACTCT
*zpd*-T7	ISH	TAATACGACTCACTATAGGGATCTGAATGGTGGCGTCGAG
*zpax3-1*-SP6	ISH	ATTTAGGTGACACTATAGAAGAGCGTCTGACACCAGAACT
*zpax3-1*-T7	ISH	TAATACGACTCACTATAGGGAGCTTGAGTCTTCGTGCTGAA
*zpc7-1*-SP6	ISH	ATTTAGGTGACACTATAGAACCTTGAAAATGGGTGCCTGC
*zpc7-1*-T7	ISH	TAATACGACTCACTATAGGGCCTCAACCTGGTCTTGAGGTC
*zpax1*-SP6	ISH	ATTTAGGTGACACTATAGAATCTGTTGAATATACAGTCCCAGAGG
*zpax1*-T7	ISH	TAATACGACTCACTATAGGGAGCTGAAGTGTGTGCGGTTA
sex-F	Gender detection	CCTAAATGATGGATGTAGATTCTGTC
sex-R	Gender detection	GATCCAGAGAAAATAAACCCAGG
*gdf9*-q-F	qPCR	ACGCTCACCTTCTGAGTTCC
*gdf9*-q-R	qPCR	AAGATCATGACGCTCAGGGG
*adgrl1*-q-F	qPCR	GTGGAAAGGCCGTGTCCTAA
*adgrl1*-q-R	qPCR	CTATTTGGCTGACCCACGGT
*rbl*-q-F	qPCR	GACGCCGTGATCAGACTACC
*rbl*-q-R	qPCR	ACATACGTAGGCCACCTCCA
*gnas*-q-F	qPCR	TGCACGCTACACTACACCTG
*gnas*-q-R	qPCR	ATGTTCTCTGTGTCGACCGC
*pou5f3*-q-F	qPCR	GCCAGAGTCGGCCATATGAT
*pou5f3*-q-R	qPCR	TAGAGGATGCTCGGGTCTGG
*adgrl1*-q-F	qPCR	CGGATGCACGAGCTTATCCT
*adgrl1*-q-R	qPCR	AGATGGGACAGGCAATGTGG
*adgrl2*-q-F	qPCR	AACGCACTCGCAACATTGTC
*adgrl2*-q-R	qPCR	TCACCCATAAGCCCCTCTCA
*ogg1*-q-F	qPCR	GGCCAAACATGCGGTACTTT
*ogg1*-q-R	qPCR	CGTGCCACATTTCCTTTCGT
*arid4b*-q-F	qPCR	CTGTGGCTGGAACGTCAGAT
*arid4b*-q-R	qPCR	GAGCGATGGACACGGTTAGT
*arid4a*-q-F	qPCR	CCTCCTCCATGTCATCGTCG
*arid4a*-q-R	qPCR	CGAGGCTTCTGGGACTTTGT

**Table A2:** List of species used in this study.

Species	Gene	Accession No
*Cynoglossus semilaevis*	Cszpax1	XP_024912764.1
	Cszpax2	XP_024912783.1
	Cszpax3-1	XP_008319235.1
	Cszpax3-2	XP_024915769.1
	Cszpax4	XP_008310879.2
	Cszpax5	XP_008319357.1
	Cszpb1a	XP_008329736.1
	Cszpb1b	XP_008328541.1
	Cszpb1c	XP_024920423.1
	Cszpb1d	XP_008315308.1
	Cszpb2a	XP_008332246.1
	Cszpb2b	XP_024912115.1
	Cszpb2c	XP_008310180.2
	Cszpc2	XP_016888570.1
	Cszpc3	XP_008323734.1
	Cszpc4a	XP_008314339.1
	Cszpc4b	XP_024914509.1
	Cszpc4c	XP_008325694.1
	Cszpc5-1	XP_008332968.3
	Cszpc5-2	NP_001284511.2
	Cszpc6	XP_008332190.1
	Cszpc7	XP_008327912.1
	Cszpc8	XP_008306734.1
	Cszpc9	XP_008325416.3
	Cszpd	XP_008309362.1
*Danio rerio*	Drzpax1	ENSDARG00000069251
	Drzpax2	ENSDARG00000017188
	Drzpax4	ENSDARG00000079034
	Drzpb2a-1	ENSDARG00000090237
	Drzpb2a-2	ENSDARG00000091409
	Drzpb2a-3	ENSDARG00000086352
	Drzpb2a-4	ENSDARG00000086522
	Drzpb2b	ENSDARG00000055415
	Drzpb2c	ENSDARG00000004898
	Drzpd	ENSDARG00000051959
	Drzpc1-1	ENSDARG00000042129
	Drzpc1-2	ENSDARG00000042130
	Drzpc2	ENSDARG00000090768
	Drzpc3	ENSDARG00000016908
	Drzpc4a-1	ENSDARG00000040081
	Drzpc4a-2	ENSDARG00000070178
	Drzpc4b	ENSDARG00000092919
	Drzpc5-1	ENSDARG00000038720
	Drzpc5-2	ENSDARG00000054313
	Drzpc6	ENSDARG00000039828
*Oreochromis niloticus*	Onzpax1	ENSONIP00000007688
	Onzpax2	ENSONIP00000007675
	Onzpax3	ENSONIP00000008459
	Onzpax4	ENSONIP00000006775
	Onzpb1a	XP_003452556.1
	Onzpb1b	ENSONIP00000010516
	Onzpb2a	ENSONIP00000012346
	Onzpb2b	ENSONIP00000018582
	Onzpb2c	ENSONIP00000018726
	Onzpd	ENSONIP00000012896
	Onzpc1	ENSONIP00000004983
	Onzpc2	ENSONIP00000018727
	Onzpc3	ENSONIP00000015344
	Onzpc4	ENSONIP00000002777
	Onzpc5-1	ENSONIP00000019557
	Onzpc5-2	ENSONIP00000019559
	Onzpc6	ENSONIP00000012513
	Onzpc7	ENSONIP00000002749
	Onzpc8	ENSONIP00000013206
*Oryzias latipes*	Olzpax1	ENSORLG00000012471
	Olzpax2	ENSORLG00000012527
	Olzpax3-1	ENSORLG00000006604
	Olzpax3-2	ENSORLG00000006621
	Olzpax4	ENSORLG00000018174
	Olzpax5	ENSORLG00000006629
	Olzpb2a	ENSORLG00000016580
	Olzpb2b	ENSORLG00000010880
	Olzpb2c	ENSORLG00000010086
	Olzpd	ENSORLG00000015608
	Olzpc1	ENSORLG00000020441
	Olzpc2	ENSORLG00000010134
	Olzpc3	ENSORLG00000005414
	Olzpc5-1	ENSORLG00000012273
	Olzpc5-2	ENSORLG00000012249
	Olzpc6	ENSORLG00000016064
	Olzpc7-1	ENSORLG00000009853
	Olzpc7-2	ENSORLG00000009867
	Olzpc8a	ENSORLG00000006875
	Olzpc8b	ENSORLG00000015033
*Pelodiscus sinensis*	Pszpa	ENSPSIG00000004165
	Pszpd	ENSPSIG00000004205
	Pszpb1	ENSPSIG00000005870
	Pszpb2a-1	ENSPSIG00000005091
	Pszpb2a-2	ENSPSIG00000014055
	Pszpb2b	ENSPSIG00000006328
	Pszpc1	ENSPSIG00000010174
	Pszpc1	ENSPSIG00000010174
	Pszpc2	ENSPSIG00000013197
	Pszpc3	ENSPSIG00000016070
	Pszpax1	ENSPSIG00000011815
	Pszpax2	ENSPSIG00000011721
*Xenopus tropicalis*	Xtzpa	ENSXETG00000016704
	Xtzpd	ENSXETG00000013767
	Xtzpb1	ENSXETG00000015911
	Xtzpb2	ENSXETG00000011855
	Xtzpc1	ENSXETG00000019465
	Xtzpc2	ENSXETG00000025433
	Xtzpax1	ENSXETG00000015572
	Xtzpax2	ENSXETG00000014953
*Gallus gallus*	Ggzpa	ENSGALP00000038302
	Ggzpb1	CAC16087.1
	Ggzpb2	ENSGALP00000032884
	Ggzpc1	ENSGALP00000002636
	Ggzpc2-1	ENSGALP00000002356
	Ggzpc2-2	ENSGALP00000002368
	Ggzpax1	ENSGALP00000026515
	Ggzpax2	ENSGALP00000026528
	Ggzpd	BAD13713.2
*Homo sapiens*	Hszpa	ENSG00000103310
	Hszpb1	ENSG00000149506
	Hszpb2	ENSG00000116996
	Hszpc	ENSG00000188372
*Latimeria chalumnae*	Lczpa	ENSLACG00000005253
	Lczpb1	ENSLACG00000005027
	Lczpb2a-1	ENSLACG00000008179
	Lczpb2a-2	ENSLACG00000007163
	Lczpb2a-3	ENSLACG00000005832
	Lczpb2a-4	ENSLACG00000004264
	Lczpb2b	ENSLACG00000011054
	Lczpb2c	ENSLACG00000007972
	Lczpc1	ENSLACG00000001864
	Lczpc2-1	ENSLACG00000000590
	Lczpc2-2	ENSLACG00000001956
	Lczpc2-3	ENSLACG00000002494
	Lczpc2-4	ENSLACG00000004624
	Lczpc5	ENSLACG00000007941
	Lczpd	ENSLACG00000002854
	Lczpax1	ENSLACG00000015100
	Lczpax2	ENSLACG00000006540

## References

[1] Xu S., Tian Z., Zhu H. (2009). Comparison of Induced and Natural Spawning in Southern Flounder *Paralichthys lethostigma*. Fish. Sci..

[2] Huang L., Liang M., Zhang H., Qu J., Wang X., Zheng K. (2013). The effect of dietary vitamin A level on reproductive performance of broodstock *Scophthamus maximus*. Prog. Fish. Sci..

[3] Bleil J.D., Wassarman P.M. (1980). Structure and function of the zona pellucida: Identification and characterization of the proteins of the mouse oocyte’s zona pellucida. Dev. Biol..

[4] Bleil J.D., Wassarman P.M. (1980). Mammalian sperm-egg interaction: Identification of a glycoprotein in mouse egg zonae pellucidae possessing receptor activity for sperm. Cell.

[5] Lefièvre L., Conner S.J., Salpekar A., Olufowobi O., Ashton P., Pavlovic B., Lenton W., Afnan M., Brewis I.A., Monk M. (2004). Four zona pellucida glycoproteins are expressed in the human. Hum. Reprod..

[6] Smith J., Paton I.R., Hughes D.C., Burt D.W. (2005). Isolation and mapping the chicken zona pellucida genes: An insight into the evolution of orthologous genes in different species. Mol. Reprod. Dev..

[7] Murata K., Sasaki T., Yasumasu S., Iuchi I., Enami J., Yasumasu I., Yamagami K. (1995). Cloning of cDNAs for the precursor protein of a low-molecular-weight subunit of the inner layer of the egg envelope (chorion) of the fish *Oryzias latipes*. Dev. Biol..

[8] Wu T., Cheng Y., Liu Z., Tao W., Zheng S., Wang D. (2018). Bioinformatic analyses of zona pellucida genes in vertebrates and their expression in Nile tilapia. Fish. Physiol. Biochem..

[9] Qi H., Williams Z., Wassarman P.M. (2002). Secretion and assembly of zona pellucida glycoproteins by growing mouse oocytes microinjected with epitope-tagged cDNAs for mZP2 and mZP3. Mol. Biol. Cell.

[10] Greve J.M., Wassarman P.M. (1985). Mouse egg extracellular coat is a matrix of interconnected filaments possessing a structural repeat. J. Mol. Biol..

[11] Bleil J.D., Greve J.M., Wassarman P.M. (1988). Identification of a secondary sperm receptor in the mouse egg zona pellucida: Role in maintenance of binding of acrosome-reacted sperm to eggs. Dev. Biol..

[12] Gao L.L., Zhou C.X., Zhang X.L., Liu P., Jin Z., Zhu G.Y., Ma Y., Li J., Yang Z.X., Zhang D. (2017). ZP3 is Required for Germinal Vesicle Breakdown in Mouse Oocyte Meiosis. Sci. Rep..

[13] Yokokawa R., Watanabe K., Kanda S., Nishino Y., Yasumasu S., Sano K. (2023). Egg envelope formation of medaka *Oryzias latipes* requires ZP proteins originating from both the liver and ovary. J. Biol. Chem..

[14] Cao Y.-Q., Wang Y.-X., Zhao Y., Zhang J., He X., Xie P., Chen J., Sun Y.-H. (2023). Transfer of the zp3a gene results in changes in egg adhesiveness and buoyancy in transgenic zebrafish. Zool. Res..

[15] Cao L., Huang Q., Wu Z., Cao D.-d., Ma Z., Xu Q., Hu P., Fu Y., Shen Y., Chan J. (2016). Neofunctionalization of zona pellucida proteins enhances freeze-prevention in the eggs of Antarctic notothenioids. Nat. Commun..

[16] Li Y., Liu Y., Fan C., Wang T., Chen C., Li Z., Li Q., Chen S. (2013). Study on the method of quick cultivation of female parental half-smooth tongue sole (*Cynoglossus semilaevis*) with nereid. J. Shanghai Ocean Univ..

[17] Wang N., Yang Q., Wang J., Shi R., Li M., Gao J., Xu W., Yang Y., Chen Y., Chen S. (2021). Integration of Transcriptome and Methylome Highlights the Roles of Cell Cycle and Hippo Signaling Pathway in Flatfish Sexual Size Dimorphism. Front. Cell Dev. Biol..

[18] Jaillon O., Aury J.-M., Brunet F., Petit J.-L., Stange-Thomann N., Mauceli E., Bouneau L., Fischer C., Ozouf-Costaz C., Bernot A. (2004). Genome duplication in the teleost fish Tetraodon nigroviridis reveals the early vertebrate proto-karyotype. Nature.

[19] Kasahara M., Naruse K., Sasaki S., Nakatani Y., Qu W., Ahsan B., Yamada T., Nagayasu Y., Doi K., Kasai Y. (2007). The medaka draft genome and insights into vertebrate genome evolution. Nature.

[20] Conner S.J., Hughes D.C. (2003). Analysis of fish ZP1/ZPB homologous genes—Evidence for both genome duplication and species-specific amplification models of evolution. Reproduction.

[21] Goudet G., Mugnier S., Callebaut I., Monget P. (2008). Phylogenetic analysis and identification of pseudogenes reveal a progressive loss of zona pellucida genes during evolution of vertebrates. Biol. Reprod..

[22] Sano K., Kawaguchi M., Yoshikawa M., Iuchi I., Yasumasu S. (2010). Evolution of the teleostean zona pellucida gene inferred from the egg envelope protein genes of the Japanese eel, *Anguilla japonica*. FEBS J..

[23] Sun Y., Yu H., Zhang Q., Qi J., Zhong Q., Chen Y., Li C. (2010). Molecular characterization and expression pattern of two zona pellucida genes in half-smooth tongue sole (*Cynoglossus semilaevis*). Comp. Biochem. Physiol. B Biochem. Mol. Biol..

[24] Braun B.C., Ringleb J., Waurich R., Viertel D., Jewgenow K. (2009). Functional role of feline zona pellucida protein 4 trefoil domain: A sperm receptor or structural component of the domestic cat zona pellucida?. Reprod. Domest. Anim..

[25] Sano K., Kawaguchi M., Katano K., Tomita K., Inokuchi M., Nagasawa T., Hiroi J., Kaneko T., Kitagawa T., Fujimoto T. (2017). Comparison of Egg Envelope Thickness in Teleosts and its Relationship to the Sites of ZP Protein Synthesis. J. Exp. Zool. B Mol. Dev. Evol..

[26] Yamagami K., Hamazaki T.S., Yasumasu S., Masuda K., Iuchi I. (1992). Molecular and cellular basis of formation, hardening, and breakdown of the egg envelope in fish. Int. Rev. Cytol..

[27] Kudo S. (2000). Enzymes responsible for the bactericidal effect in extracts of vitelline and fertilisation envelopes of rainbow trout eggs. Zygote.

[28] Murata K., Sugiyama H., Yasumasu S., Iuchi I., Yasumasu I., Yamagami K. (1997). Cloning of cDNA and estrogen-induced hepatic gene expression for choriogenin H, a precursor protein of the fish egg envelope (chorion). Proc. Natl. Acad. Sci. USA.

[29] Hyllner S.J., Westerlund L., Olsson P.E., Schopen A. (2001). Cloning of rainbow trout egg envelope proteins: Members of a unique group of structural proteins. Biol. Reprod..

[30] Wang H., Gong Z. (1999). Characterization of two zebrafish cDNA clones encoding egg envelope proteins ZP2 and ZP3. Biochim. Biophys. Acta.

[31] Hasuwa H., Ueda J., Ikawa M., Okabe M. (2013). miR-200b and miR-429 function in mouse ovulation and are essential for female fertility. Science.

[32] Liu X., Huang Y., Tan F., Wang H.Y., Chen J.Y., Zhang X., Zhao X., Liu K., Wang Q., Liu S. (2022). Single-Cell Atlas of the Chinese Tongue Sole (*Cynoglossus semilaevis*) Ovary Reveals Transcriptional Programs of Oogenesis in Fish. Front. Cell Dev. Biol..

[33] Nosek J., Krajhanzl A., Kocourek J. (1983). Studies on lectins. LVII. Immunofluorescence localization of lectins present in fish ovaries. Histochemistry.

[34] Dong C.-H., Yang S.-T., Yang Z.-A., Zhang L., Gui J.-F. (2004). A C-type lectin associated and translocated with cortical granules during oocyte maturation and egg fertilization in fish. Dev. Biol..

[35] Zhou J., Sun S., Li R., Xu H., Li M., Li Z. (2024). Transcriptome analysis of *Schizothorax oconnori* (Cypriniformes: Cyprinidae) oocytes: The role of K(+) in promoting yolk globule fusion and regulating oocyte maturation. Fish. Physiol. Biochem..

[36] Mukherjee D., Majumder S., Roy Moulik S., Pal P., Gupta S., Guha P., Kumar D. (2017). Membrane receptor cross talk in gonadotropin-, IGF-I-, and insulin-mediated steroidogenesis in fish ovary: An overview. Gen. Comp. Endocrinol..

[37] França M.M., Mendonca B.B. (2022). Genetics of ovarian insufficiency and defects of folliculogenesis. Best. Pract. Res. Clin. Endocrinol. Metab..

[38] Ren D., Wei X., Lin L., Yuan F., Bi Y., Guo Z., Liu L., Ji L., Yang X., Han K. (2022). A novel heterozygous missense variant of the ARID4A gene identified in Han Chinese families with schizophrenia-diagnosed siblings that interferes with DNA-binding activity. Mol. Psychiatry.

[39] Wu R.C., Jiang M., Beaudet A.L., Wu M.Y. (2013). ARID4A and ARID4B regulate male fertility, a functional link to the AR and RB pathways. Proc. Natl. Acad. Sci. USA.

[40] Zuccotti M., Merico V., Sacchi L., Bellone M., Brink T.C., Bellazzi R., Stefanelli M., Redi C.A., Garagna S., Adjaye J. (2008). Maternal Oct-4 is a potential key regulator of the developmental competence of mouse oocytes. BMC Dev. Biol..

[41] Sánchez-Sánchez A.V., Camp E., García-España A., Leal-Tassias A., Mullor J.L. (2010). Medaka Oct4 is expressed during early embryo development, and in primordial germ cells and adult gonads. Dev. Dyn..

[42] Paulini F., Melo E.O. (2011). The role of oocyte-secreted factors GDF9 and BMP15 in follicular development and oogenesis. Reprod. Domest. Anim..

[43] Shi R., Li X., Cheng P., Yang Q., Chen Z., Chen S., Wang N. (2022). Characterization of growth differentiation factor 9 and bone morphogenetic factor 15 in Chinese tongue sole (*Cynoglossus semilaevis*): Sex-biased expression pattern and promoter regulation. Theriogenology.

[44] Lord T., Aitken R.J. (2015). Fertilization stimulates 8-hydroxy-2′-deoxyguanosine repair and antioxidant activity to prevent mutagenesis in the embryo. Dev. Biol..

[45] Young M., McPhaul M.J. (1998). A steroidogenic factor-1-binding site and cyclic adenosine 3′,5′-monophosphate response element-like elements are required for the activity of the rat aromatase promoter in rat Leydig tumor cell lines. Endocrinology.

[46] Shao C., Liu G., Liu S., Liu C., Chen S. (2013). Characterization of the cyp19a1a gene from a BAC sequence in half-smooth tongue sole (*Cynoglossus semilaevis*) and analysis of its conservation among teleosts. Acta Oceanol. Sin..

[47] Zhang C., He Q., Cheng H., Li L., Ruan X., Duan X., Huang F., Yang H., Zhang H., Shi H. (2022). Transcription factors foxl2 and foxl3 regulate cyp19a1a and cyp11b in orange-spotted grouper (*Epinephelus coioides*). Aquac. Rep..

[48] Yang Y.J., Wang Y., Li Z., Zhou L., Gui J.F. (2017). Sequential, Divergent, and Cooperative Requirements of Foxl2a and Foxl2b in Ovary Development and Maintenance of Zebrafish. Genetics.

[49] Xu X., Wang X., Zhou L., Liu Q., Li J. (2021). Changes of estrogen, aromatase and estrogen receptor during oogenesis and gestation of *Sebastes schlegelii*. Oceanol. Limnol. Sin..

[50] Dong J., Albertini D.F., Nishimori K., Kumar T.R., Lu N., Matzuk M.M. (1996). Growth differentiation factor-9 is required during early ovarian folliculogenesis. Nature.

[51] Curry T.E. (2010). ADAMTS1 and versican: Partners in ovulation and fertilization. Biol. Reprod..

[52] Shozu M., Minami N., Yokoyama H., Inoue M., Kurihara H., Matsushima K., Kuno K. (2005). ADAMTS-1 is involved in normal follicular development, ovulatory process and organization of the medullary vascular network in the ovary. J. Mol. Endocrinol..

[53] Brown H.M., Dunning K.R., Robker R.L., Pritchard M., Russell D.L. (2006). Requirement for ADAMTS-1 in extracellular matrix remodeling during ovarian folliculogenesis and lymphangiogenesis. Dev. Biol..

[54] Rankin T.L., O’Brien M., Lee E., Wigglesworth K., Eppig J., Dean J. (2001). Defective zonae pellucidae in Zp2-null mice disrupt folliculogenesis, fertility and development. Development.

[55] Liu W., Li K., Bai D., Yin J., Tang Y., Chi F., Zhang L., Wang Y., Pan J., Liang S. (2017). Dosage effects of ZP2 and ZP3 heterozygous mutations cause human infertility. Hum. Genet..

[56] Rankin T., Talbot P., Lee E., Dean J. (1999). Abnormal zonae pellucidae in mice lacking ZP1 result in early embryonic loss. Development.

[57] Lamas-Toranzo I., Fonseca Balvís N., Querejeta-Fernández A., Izquierdo-Rico M.J., González-Brusi L., Lorenzo P.L., García-Rebollar P., Avilés M., Bermejo-Álvarez P. (2019). ZP4 confers structural properties to the zona pellucida essential for embryo development. eLife.

[58] Liu Y., Chen S., Gao F., Meng L., Hu Q., Song W., Shao C., Lv W. (2014). SCAR-transformation of Sex-specific SSR Marker and Its Application inHalf-smooth Tongue Sole (*Cynoglossus semiliaevis*). J. Agric. Biotechnol..

[59] Mistry J., Chuguransky S., Williams L., Qureshi M., Salazar Gustavo A., Sonnhammer E.L.L., Tosatto S.C.E., Paladin L., Raj S., Richardson L.J. (2020). Pfam: The protein families database in 2021. Nucleic Acids Res..

[60] Eddy S.R. (1996). Hidden Markov models. Curr. Opin. Struct. Biol..

[61] Altschul S.F., Gish W., Miller W., Myers E.W., Lipman D.J. (1990). Basic local alignment search tool. J. Mol. Biol..

[62] Camacho C., Coulouris G., Avagyan V., Ma N., Papadopoulos J., Bealer K., Madden T.L. (2009). BLAST+: Architecture and applications. BMC Bioinform..

[63] Katoh K., Standley D.M., Russell D.J. (2014). MAFFT: Iterative Refinement and Additional Methods. Multiple Sequence Alignment Methods.

[64] Minh B.Q., Schmidt H.A., Chernomor O., Schrempf D., Woodhams M.D., von Haeseler A., Lanfear R. (2020). IQ-TREE 2: New Models and Efficient Methods for Phylogenetic Inference in the Genomic Era. Mol. Biol. Evol..

[65] Letunic I., Bork P. (2021). Interactive Tree Of Life (iTOL) v5: An online tool for phylogenetic tree display and annotation. Nucleic Acids Res..

[66] Hu B., Jin J., Guo A.-Y., Zhang H., Luo J., Gao G. (2014). GSDS 2.0: An upgraded gene feature visualization server. Bioinformatics.

[67] Bailey T.L., Boden M., Buske F.A., Frith M., Grant C.E., Clementi L., Ren J., Li W.W., Noble W.S. (2009). MEME Suite: Tools for motif discovery and searching. Nucleic Acids Res..

[68] Chen C., Chen H., Zhang Y., Thomas H.R., Frank M.H., He Y., Xia R. (2020). TBtools: An Integrative Toolkit Developed for Interactive Analyses of Big Biological Data. Mol. Plant.

[69] Louis A., Muffato M., Roest Crollius H. (2012). Genomicus: Five genome browsers for comparative genomics in eukaryota. Nucleic Acids Res..

[70] Zhu Y., Li S., Su B., Xue T., Cao M., Li C. (2021). Genome-wide identification, characterization, and expression of the Toll-like receptors in Japanese flounder (*Paralichthys olivaceus*). Aquaculture.

[71] Szklarczyk D., Kirsch R., Koutrouli M., Nastou K., Mehryary F., Hachilif R., Gable A.L., Fang T., Doncheva N.T., Pyysalo S. (2022). The STRING database in 2023: Protein–protein association networks and functional enrichment analyses for any sequenced genome of interest. Nucleic Acids Res..

[72] Livak K.J., Schmittgen T.D. (2001). Analysis of Relative Gene Expression Data Using Real-Time Quantitative PCR and the 2^−ΔΔCT^ Method. Methods.

[73] Zhu Y., Cui Z., Yang Y., Xu W., Shao C., Fu X., Li Y., Chen S. (2019). Expression analysis and characterization of dmrt2 in Chinese tongue sole (*Cynoglossus semilaevis*). Theriogenology.

